# Association between dietary patterns and metabolic syndrome in Chinese adults: a propensity score-matched case-control study

**DOI:** 10.1038/srep34748

**Published:** 2016-10-06

**Authors:** Yang Xia, Yeqing Gu, Fei Yu, Qing Zhang, Li Liu, Ge Meng, Hongmei Wu, Huanmin Du, Hongbin Shi, Xiaoyan Guo, Xing Liu, Chunlei Li, Peipei Han, Renwei Dong, Xiuyang Wang, Xue Bao, Qian Su, Liyun Fang, Fangfang Liu, Huijun Yang, Li Kang, Yixuan Ma, Bin Yu, Shaomei Sun, Xing Wang, Ming Zhou, Qiyu Jia, Qi Guo, Yuntang Wu, Kun Song, Guowei Huang, Guolin Wang, Kaijun Niu

**Affiliations:** 1 Nutritional Epidemiology Institute and School of Public Health, Tianjin Medical University, Tianjin, China; 2 Health Management Centre, Tianjin Medical University General Hospital, Tianjin, China; 3 Department of Rehabilitation and Sports Medicine, Tianjin Medical University, Tianjin, China; 4 Collaborative Innovation Center of Non-communicable Disease, Tianjin Medical University, Tianjin, China

## Abstract

Previous studies indicated that dietary patterns were associated with metabolic syndrome (MS), but little is known in Chinese. We design this case-control study to evaluate the associations between dietary patterns and MS in Chinese adults. In this study, 1492 participants with MS were matched with 1492 controls using the 1:1 ratio propensity score matching methods. Dietary intake was assessed using a valid self-administered food frequency questionnaire, and MS was defined in accordance with the criteria of the American Heart Association scientific statement of 2009. Higher scores for the high-protein/cholesterol pattern were associated with higher prevalence of MS. Compared with the participants in the lowest quartile, the odds ratio (OR) for the extreme quartile was 1.36 (95% confidence interval (CI), 1.10–1.68) and the *P* for trend <0.01 after adjusted for the other two dietary pattern scores. We also found a moderate consumption of the balanced pattern was associated with the lowest prevalence of MS. The ORs across quartiles of the balanced pattern were 1 (reference), 0.83 (95% CI, 0.68–1.02), 0.69 (95% CI, 0.56–0.85), and 0.84 (95% CI, 0.68–1.04) after adjustment. Our study demonstrates that there is a strong association between a diet rich in animal offal, animal blood, meat, and sausage and a higher prevalence of MS.

Metabolic syndrome (MS) is a cluster of cardiovascular risk factor abnormalities and the prevalence of MS is increasing rapidly throughout the world, in parallel with the increased risk of type 2 diabetes mellitus^1^, cardiovascular disease (CVD)^2^, and all-cause mortality^3^. The prevalence of MS in China was reported vary from 7.3% to 31.5% in previous studies conducted in different parts^4^,^5^,^6^ due to the different definitions and regional differences.

Unlike studies focused on the effect of single food item and nutrient, examination of dietary patterns, which assess the effects of overall diet and determined by factor analysis can be proxy indicators of real food consumption and availability, providing a more realistic representation of everyday eating habits^7^. Several studies have demonstrated that dietary pattern is an important factor associated with MS^8^,^9^,^10^,^11^,^12^,^13^. There are three cross-sectional studies explored the association between dietary pattern and prevalence of MS in Chinese^14^,^15^,^16^. A cross-sectional study from China National Nutrition and Health Survey demonstrated that the ‘Green Water’ dietary pattern, characterized by high intakes of rice and vegetables and moderate intakes in animal foods was related to the lowest prevalence of MS (15.9%). Compared to the ‘Green Water’ dietary pattern, the ‘Yellow Earth’ dietary pattern, characterized by high intakes of refined cereal products, tubers, cooking salt and salted vegetable was associated with a significantly elevated odds of MS (OR: 1.66, 95% CI: 1.40–1.96). The ‘Western/new affluence’ dietary pattern characterized by higher consumption of beef/lamb, fruit, eggs, poultry and seafood also significantly associated with MS (OR: 1.37, 95% CI: 1.13–1.67)^14^. A limitation of this study was the lack of validation of the physical activity questionnaire^14^. Another cross-sectional study in Chinese found the ‘Dairy/Eggs’ pattern was associated with a decreased odds of MS only in women (OR: 0.45, 95% CI: 0.26–0.79) and no significant association between other patterns and MS^15^. However, the adjustment was incomplete in this study (only age, occupation, types of area, and body mass index were adjusted)^15^. Considering the design of cross-sectional study, it is disallows a sequence of temporality to be established for MS and dietary patterns. Therefore we designed this case-control study to determine the association between dietary patterns and the prevalence of MS among Chinese adults.

## Results

### Dietary patterns

After varimax rotation, factor analysis revealed three dietary patterns and the main factor loadings of each pattern (Table 1). The three dietary patterns accounted for 27.4% of the variance in total food intake. According to the contribution to the total variation, the three dietary patterns were: factor one was defined as the high-carbohydrate/sweet pattern and characterized by high intake of candied fruits, cakes, ice cream, and juice; factor two, the balanced pattern, was typified by a balance intake of vegetables, mushroom and coarse cereals; factor three, identified as a high-protein/cholesterol pattern and included high intakes of animal offal, animal blood, and sausage.

### Characteristics of participants

Characteristics of participants according to MS status before and after propensity scores matching are shown in Tables 2 and 3, respectively.

Among 8313 participants who were available to be analyzed before propensity score matching, 19.68% were classified as newly diagnosed MS. As shown in Table 2, participants with MS trended to be men (*P* < 0.0001), older (*P* < 0.0001), current smoker (*P* < 0.0001) or ex-smoker (*P* < 0.0001), drinking everyday (*P* < 0.0001), who had higher BMI (*P* < 0.0001), lower education level (*P* < 0.0001), lower household income (*P* < 0.01), a family history of hyperlipidemia (*P* = 0.048), and employ as managers (*P* < 0.0001) or professionals (*P* < 0.0001). After propensity score matching, 1492 cases and 1492 controls were generated and showed no significant baseline differences in any character (Table 3).

### Dietary patterns and metabolic syndrome

Higher scores for the high-protein/cholesterol pattern were associated with higher prevalence of MS (Table 4). The OR for the extreme quartile is 1.36 (95% CI, 1.10–1.68) and the *P* for trend <0.01 after adjusted for the other two dietary pattern scores. A moderate consumption of the balanced pattern was associated with the lowest prevalence of MS. The ORs across quartiles of balanced pattern were 1 (reference), 0.83 (95% CI, 0.68–1.02), 0.69 (95% CI, 0.56–0.85), and 0.84 (95% CI, 0.68–1.04) after adjustment.

Results of major food groups’ analyses were presented in Table 5. Consumption of animal foods was associated with higher prevalence of MS, the OR for the extreme quartile was 1.21 (95% CI, 1.01–1.51) after adjustment. A moderate consumption of vegetables was associated with the lowest prevalence of MS. The ORs across quartiles of consumption of vegetables were 1 (reference), 0.79 (95% CI, 0.64–0.97), 0.67 (95% CI, 0.54–0.83), and 0.83 (95% CI, 0.65–1.05) after adjustment for intake of other food groups.

## Discussion

A former cross-sectional study based on this dynamic cohort used data collected from May 2013 to December 2013 (4365 participants were included in the statistical analysis finally) and assessed the associations between dietary patterns and fatty liver disease in adults firstly^17^. Results suggested that the high-protein/cholesterol pattern scores are associated with higher prevalence of alcoholic fatty liver disease in males and the high-carbohydrate/sweet pattern scores are associated with higher prevalence of non-alcoholic fatty liver disease in females^17^. In the present case-control study, data collected from May 2013 to December 2014 (8313 participants were included in the statistical analysis finally) were used to explore the associations between dietary patterns and MS. The characteristics of populations and dietary patterns identified in these two studies are similar. Compared with the former study^17^, the sample size of the present study is bigger and more potential confounders were adjusted. Moreover, we used the propensity score matching method, which is convenient for matching when a lot of covariates must be taken into consideration, to generate cases and controls. Compared with adjustments in the regression model, the propensity score matching method is useful in determining whether the covariates have been correctly specified^18^.

In order to generate the control samples for cases, we used the propensity scores matching method, which is increasingly been used to offer investigators the ability to balance treated groups across all putative risk factors and allows easy inspection of the achieved balance across measured covariates^19^. Therefore, the associations between dietary patterns and MS were independent of confounding factors as the propensity scores matching approach reduced the differences between the case group and the control group. Three main dietary patterns were identified among adults in Tianjin, including a high-carbohydrate/sweet pattern, a balanced pattern, and a high-protein/cholesterol pattern.

Results suggested that there is a strong association between high-protein/cholesterol pattern as characterized by high intakes of animal offal, animal blood, and sausage and the higher prevalence of MS. This finding was in line with previous studies, which demonstrated that the Western dietary pattern was associated with a higher prevalence of MS^8^,^12^. Another cross-sectional study conducted in South Korea also showed that the meat dietary pattern was strongly correlated with the occurrence of MS^13^. There are several plausible mechanisms underlying this association. First, these kinds of dietary patterns contain a lot of fat, especially saturated fat, and high energy-dense foods. Consumption of saturated fat was associated with MS risk factors, such as blood pressure^20^ and dyslipemia^21^. Second, red meat and animal blood were related to the deposition of iron while iron overload was positively associated with insulin resistance^22^; a previous study showed that MS subjects had a significantly higher prevalence of iron overload than control subjects^23^. Third, meat intake was associated with inflammation^24^; and a chronic state of inflammation appears to be a central mechanism underlying the pathophysiology of MS^25^; previous studies showed that inflammation markers, such as interleukin-6^26^ and C-reactive protein^27^ were positively associated with MS. Fourth, Zn from red meat, but not from other sources, was associated with a risk of MS. This association was supported by findings from the Supplementation en Vitamines et Minéraux AntioXydants Study, showing a positive association between serum Zn concentration and MS^28^. Furthermore, the major food group analyses confirm this association. In line with previous studies^8^,^29^, our results suggest that there is a strong association between the consumption of animal foods, especially meat, and the higher prevalence of MS.

We also observed a negative association between consumption of the balanced pattern, which has been characterized by intake of vegetables, coarse cereals and fruits, and the prevalence of MS. A previous study demonstrated the health dietary pattern with a high intake of vegetables, fruits, fish, and seafood was inversely associated with the prevalence of MS^9^. In this study, there was a decrease of prevalence in the third quartile (OR, 0.69; 95% CI, 0.56–0.85) of the balanced pattern, however this association turned to be non-significant in the fourth quartile (OR, 0.84; 95% CI, 0.68–1.04). The results suggested that the association between this dietary pattern and MS may be non-linear. We then conducted the analyses of the major components of this dietary pattern and MS. Participants consuming 392–554 gram of vegetables per day were less likely to report MS compared with participants who had an intake of 0–274 gram of vegetables per day (OR, 0.67; 95% CI, 0.54–0.83) after adjustment for the intake of other food groups. This association turned to be non-significant when participants’ consumption of vegetables was higher than 554 grams/day. Meanwhile, the consumption of 99–162 gram of fruits per day showed the lowest OR on MS even though it was not statistically significant. A study conducted in Korea found that compared with the bottom quintile, the multivariable-adjusted risk ratio (95% CI) for individuals across quintiles of the vegetables intake were 1 (reference), 0.98 (0.82, 1.19), 0.93 (0.76, 1.13), 0.85 (0.76, 1.13), and 0.99 (0.80, 1.22) which suggested a non-linear association between the intake of vegetables and MS even though it was not statistically significant^9^. This suggested that the association between consumption of fruits/vegetables and MS may be non-linear. The mechanisms that have been proposed to explain the non-linear associations are mainly related to the sugar content of fruit and some kinds of vegetables. A previous study suggested that increased consumption of a diet rich in sucrose, fructose, or glucose, is responsible for the increasing incidence of obesity, T2DM, and MS^30^. In the liver, fructose bypasses that whole machinery because it does not need phosphofructokinase. Furthermore, most, if not all, of the fructose that is consumed gets converted to fat^30^. Thus a high intake of fructose was associated with the prevalence of MS^30^ even though fruits and vegetables contain amounts of beta carotene, vitamins C and E, folate, polyphenols, and various minerals and then help prevent MS^31^. In our study, as shown in our results, a moderate intake of the balanced pattern may help prevent MS.

A few limitations are notable. First, due to the nature of the self-reporting questionnaire, recall bias exists and the food intake may be not exact. Second, we exclude participants for reason of health conditions and the final sample may not be representative of the population, while it is reasonable to exclude these participants with CVD and cancer when we estimate the association between dietary patterns and MS.

## Materials and Methods

### Participants

This case-control study is based on the Tianjin Chronic Low-grade Systemic Inflammation and Health (TCLSIHealth) Cohort Study, which is a large prospective dynamic cohort study focusing on the relationships between chronic low-grade systemic inflammation and the healthy status of a population living in Tianjin, China^32^. Participants were recruited while having their annual health examinations at the Tianjin Medical University General Hospital-Health Management Center, the largest and most comprehensive physical examination center in Tianjin. This dynamic cohort study was launched in 2007. Moreover, a detailed lifestyle questionnaire covering family income, marital status, employment status, educational level, physical activity, sleep habits, dietary habits, overall computer/mobile device usage time, television time, history of prior infections, and use of medicines as well as physical performance tests were administered to randomly selected subjects from this population since May 2013. A former study based on this cohort used data collected from May 2013 to December 2013^17^. The present study used data collected from May 2013 to December 2014. Ethical approval was given by the medical ethics committee of Institutional Review Board of the Tianjin Medical University with the reference number of TMUhMEC 201430 and participants gave written informed consent prior to participation in the study. The methods of this study were carried out in accordance with the approved guidelines.

Seventeen thousand two hundred and fifty-seven participants completed a comprehensive health examination, including evaluation of anthropometric parameters, biochemical blood examination (included fasting blood glucose (FBG), triglycerides (TG), and high-density lipoprotein cholesterol (HDL) etc.), and collection of blood samples for follow-up experiments from 2013 to 2014. Participants also completed a study questionnaire reporting personal information, dietary intake, lifestyle and health condition. We excluded participants who did not provide information for assessment of MS (n = 2016), did not provide information of diets (n = 861), body mass index (BMI) (n = 6), and physical activity (n = 27), had changed their lifestyles in last 5 years (n = 3143), had been diagnosed with CVD (n = 747), cancer (n = 204), and diabetes (n = 717). We also excluded participants had a history of MS (n = 1223). The final study population comprised 8313 (1636 cases, 6677 controls) participants for propensity score matching.

### Propensity score matching

Propensity score were calculated using a logistic regression model and the following covariates: sex, age, BMI, physical activity, energy intake, education level, household income level, birth in local, living time in local, smoking status, drinking status, employment status, and family history of cardiovascular disease, hypertension, hyperlipidemia and diabetes. Using these propensity scores, cases were individually matched by controls using the nearest matching method within a caliper distance, which selects for matching a control subject whose propensity score is closest to that of the case subject (nearest neighbor matching approach) with the further restriction that the absolute difference in the propensity scores of matched subjects must be below some pre-specified threshould (the caliper distance)^18^. Thus, participants for whom the propensity score could not be matched because of a greater caliper distance were excluded from further analysis. As suggested by Austin^18^, a caliper of width equal to 0.2 of the standard deviation of the logit of the propensity score was used, as this value minimized the mean squared error of the estimated treatment effect in several scenarios. To better match cases and controls, we used the 1:1 ratio matching method. If a case subject could not be matched to any control subject, then the case subject was discarded. Finally, 1492 cases and 1492 controls were generated using this propensity score matching method.

### Identification of dietary patterns

Dietary intake was assessed using an evaluated semi-quantitative food frequency questionnaire (FFQ). The FFQ consisted of 81 items, including 7 frequency categories ranging from ‘almost never eat’ to ‘twice or more per day’ for foods and 8 frequency categories ranging from ‘almost never drink’ to ‘four or more times per day’ for beverages. The reproducibility of the questionnaire were assessed in a random sample of 150 participants and living in Tianjin by comparing the data from the questionnaire with the data from 2 dietary questionnaires collected approximately 3 months apart. Spearman rank correlation coefficient for energy intake between 2 food frequency questionnaires administered 3 months apart was 0.67. Spearman’s rank correlation coefficient for energy intake by the 4-day dietary records (DRs) and the second FFQ was 0.58. We applied factor analysis in order to generate major dietary patterns and factor loadings on all 81 food items and beverages. Varimax rotation was applied for greater interpretability. After evaluation of eigenvalues (greater than 1.0) and the scree test, 3 factors were determined. Food items with a factor loading greater than |0.30| were the main contributors to dietary pattern and representative of the character of each pattern. Factors were named descriptively according to the food items showing high loading (absolute value) with respect to each dietary pattern as follows: ‘high-carbohydrate/sweet’ pattern, ‘balanced’ pattern, and ‘high-protein/cholesterol’ pattern.

### Assessment of metabolic syndrome

Waist circumference was measured at the umbilical level with participants standing and breathing normally. Blood pressure (BP) was measured twice from the upper left arm using a TM-2655P automatic device (A&D CO., Tokyo, Japan) after 5 minutes of rest in a seated position. The mean of these 2 measurements was taken as the BP value. Blood samples for the analysis of fasting blood glucose (FBG) and lipids were collected in siliconized vacuum plastic tubes. FBS was measured by the glucose oxidase method, triglycerides (TG) were measured by enzymatic methods, low-density lipoprotein cholesterol (LDL) was measured by the polyvinyl sulfuric acid precipitation method, and high-density lipoprotein cholesterol (HDL) was measured by the chemical precipitation method using reagents from Roche Diagnostics on an automatic biochemistry analyzer (Roche Cobas 8000 modular analyzer, Mannheim, Germany).

Metabolic syndrome was defined in accordance with the criteria of the American Heart Association scientific statement of 2009^33^. Participants were considered to have MS when they presented three or more of the following components: 1) elevated waist circumference for Chinese individuals (≥85 cm in males; ≥80 cm in females), 2) elevated TG (≥1.7 mmol/L), or drug treatment for elevated TG, 3) reduced HDL (<1.0 mmol/L in males; <1.3 mmol/L in females) or drug treatment for reduced HDL, 4) elevated blood pressure (SBP ≥130 mm Hg and/or DBP ≥85 mm Hg) or antihypertensive drug treatment, 5) elevated fasting glucose (≥5.56 mmol/L) or drug treatment for elevated glucose.

### Assessment of other variables

The sociodemographic variables, which include sex, age, education, employment, and income, were also assessed. The educational level was assessed by asking the question “what is the highest degree you earned?” and was divided into 2 categories: <College graduate or ≥College graduate. Employment status was classified as either Senior Officials and Managers or Professionals. Information on the smoking (“never,” “former,” and “current smoking”) and drinking (“never,” “former,” and “current drinking”) status of the participants was obtained from a questionnaire survey. Living time in local was divided into 2 categories: <10 years or ≥10 years. The subjects were also classified as birth in local or not. Physical activity (PA) in the most recent week was assessed using the short form of the International Physical Activity Questionnaire (IPAQ)^34^. The questionnaire asked whether subjects had performed any activities from the following categories during the previous week: walking; moderate activity (household activity or child care); vigorous activity (running, swimming, or other sports activities). Metabolic equivalent (MET) hours per week were calculated using corresponding MET coefficients (3.3, 4.0 and 8.0, respectively) according to the following formula: MET coefficient of activity × duration (hours) × frequency (days). Total PA levels were assessed by combining separate scores for different activities.

### Statistical analysis

Descriptive data have been presented as the least square mean (with 95% CI) and examined using analysis of variance according to MS status. Categorical variables have been presented as percentages and examined using chi-square test according to MS status. Quartiles were categorized across the scores of each dietary pattern based on the distribution of the scores for all the participants and used for further analyses. Association between quartilecategories of dietary pattern scores and MS status were examined using conditional logistic regression analysis. MS status was used as dependent variable, and factor score was used as independent variable. OR and 95% CI were calculated. A linear trend cross increasing quartiles was tested using the median value of each quartile as a continuous variable based on linear regression. Model 1 was used to calculate the crude OR and model 2 additionally adjusted for other dietary pattern factor scores or intake of food group with each other. All analyses were performed using the Statistical Analysis System 9.3 edition for Windows (SAS Institute Inc., Cary, NC, USA) and STATA (version 12.1; Stata Corp LP, College Station, TX, USA). All *P*-values were two-tailed and difference was defined to be significant when *P* < 0.05.

## Additional Information

**How to cite this article**: Xia, Y. *et al*. Association between dietary patterns and metabolic syndrome in Chinese adults: a propensity score-matched case-control study. *Sci. Rep*. **6**, 34748; doi: 10.1038/srep34748 (2016).

## Figures and Tables

**Table 1:** The factor loadings scores^a^ of primary food groups of dietary patterns.

high-carbohydrate/sweet pattern		balanced pattern		high-protein/cholesterol pattern	
food groups	factor loadings	food groups	factor loadings	food groups	factor loadings
pineapple	0.66	pumpkin, carrot	0.64	animal offal (except for animal liver)	0.67
strawberry, kiwi fruit, persimmon	0.66	celery	0.63	animal liver	0.66
sweets, candied fruits	0.63	Chinese cabbage	0.59	preserved egg	0.59
fruit juice, vegetable juice	0.63	cucumber	0.59	animal blood	0.58
salted eggs	0.60	Chinese watermelon	0.58	sausage	0.58
ice cream	0.60	eggplant	0.56	pork skin	0.58
western-style pastry, cakes	0.60	tomato (including the ketchup)	0.54	instant noodle	0.53
white wine	0.59	apple	0.54	wonton	0.52
preserved bean curd	0.58	radish (expect for carrot)	0.53	sea fish	0.49
cookies	0.57	green vegetable	0.52	freshwater fish	0.48
peach	0.57	sweet potato	0.50	seafood (shellfish, squid, shrimp)	0.47
Chinese cakes	0.57	coarse cereals	0.50	miscellaneous sauce noodles	0.46
Chinese sauerkraut	0.56	potato (except for sweet potato)	0.50	Poultry	0.46
grape	0.56	onion	0.48	steamed stuffed bun, dumpling	0.46
carbonated beverage	0.55	mushroom	0.46	Meat	0.39
watermelon	0.55	congee	0.46	Bread	0.38
other kinds of fruit	0.51	bell peppers	0.45	Chinese watermelon	0.36
pear	0.51	soya bean products	0.45	Beer	0.35
coffee	0.48	lotus root	0.45	Eggplant	0.33
leek	0.48	pear	0.44	low-fat milk	0.33

^a^For simplicity, only the top 20 food groups of factor loading scores of each pattern are shown.

**Table 2:** Participant characteristics by metabolic syndrome status before matching.

Characteristics	Metabolic syndrome status (Before matching)		*P* value^a^
	No (n = 6677)	Yes (n = 1636)	
Sex (male, %)	47.73	68.22	<0.0001
Age (y)	41.55 (41.28, 41.82)^b^	47.37 (46.75, 47.99)	<0.0001
BMI (kg/m^2^)	23.41 (23.34, 23.48)	27.32 (27.15, 27.49)	<0.0001
Physical activity (Mets × hours/week)	9.08 (8.77, 9.36)	9.36 (8.74, 10.03)	0.43
Energy intake (kcal/d)	2210.42 (2185.33, 2235.79)	2251.05 (2199.73, 2303.57)	0.17
Education (≥College graduate, %)	61.49	40.59	<0.0001
Household income (≥10,000 Yuan, %)	36.86	33.19	<0.01
Born in local (Yes, %)	61.18	63.69	0.06
Living time in local (≥10 years, %)	77.82	78.97	0.31
Smoking status (%)			
Smoker	16.98	21.58	<0.0001
Ex-smoker	4.42	7.46	<0.0001
Drinking status (%)			
Everyday	4.13	9.41	<0.0001
Sometime	58.18	56.54	0.23
Ex-drinker	6.80	7.03	0.74
Employment status (%)			
Managers	36.77	29.16	<0.0001
Professionals	17.22	13.33	<0.0001
Family history of diseases (%)			
CVD	30.90	29.58	0.30
Hypertension	45.92	45.17	0.59
Hyperlipidemia	0.46	0.12	0.048
Diabetes	19.05	19.80	0.49

^a^Analysis of variance or chi-square test.

^b^Least square mean (95% confidence interval) (all such values).

**Table 3:** Participant characteristics by metabolic syndrome status after matching.

Characteristics	Metabolic syndrome status (After matching)		*P* value^a^
	No (n = 1492)	Yes (n = 1492)	
Sex (male, %)	68.83	67.23	0.35
Age (y)	47.39 (46.78, 48.01)^b^	47.21 (46.60, 47.82)	0.68
BMI (kg/m^2^)	26.88 (26.74, 27.03)	26.99 (26.84, 27.13)	0.32
Physical activity (Mets × hours/week)	9.62 (8.93, 10.37)	9.50 (8.81, 10.23)	0.81
Energy intake (kcal/d)	2253.16 (2197.59, 2310.13)	2251.09 (2195.58, 2308.02)	0.96
Education (≥College graduate, %)	42.96	41.82	0.53
Household income (≥10,000 Yuan, %)	33.85	33.91	0.97
Born in local (Yes, %)	62.94	63.07	0.94
Living time in local (≥10 years, %)	79.76	78.62	0.44
Smoking status (%)			
Smoker	23.06	21.98	0.48
Ex-smoker	8.04	7.64	0.68
Drinking status (%)			
Everyday	9.05	9.12	0.95
Sometime	58.91	57.10	0.32
Ex-drinker	6.50	6.90	0.66
Employment status (%)			
Managers	31.23	30.09	0.50
Professionals	13.54	13.67	0.92
Family history of diseases (%)			
CVD	31.50	30.70	0.64
Hypertension	44.91	45.38	0.80
Hyperlipidemia	0.20	0.13	0.65
Diabetes	19.03	19.97	0.52

^a^Analysis of variance or chi-square test.

^b^Least square mean (95% confidence interval) (all such values).

**Table 4:** Association between quartiles of factor scores and metabolic syndrome.

Dietary patterns	Quartiles of factor scores (range, n = 2984)				*P* for trend^a^
High-carbohydrate/sweet pattern	Level 1 (−4.75, −0.40)	Level 2 (−0.40, −0.13)	Level 3 (−0.13, 0.19)	Level 4 (0.19, 14.61)	
No. of metabolic syndrome	372	395	351	374	
Crude	Ref	1.12 (0.92, 1.37)^b^	0.89 (0.72, 1.09)	1.01 (0.83, 1.24)	0.59
Adjusted^c^	Ref	1.14 (0.93, 1.40)	0.91 (0.74, 1.13)	1.04 (0.85, 1.28)	0.91
Balanced pattern	Level 1 (−2.73, −0.61)	Level 2 (−0.61, −0.18)	Level 3 (−0.18, 0.36)	Level 4 (0.36, 8.67)	
No. of metabolic syndrome	409	373	335	375	
Crude	Ref	0.82 (0.67, 1.01)	0.67 (0.55, 0.83)	0.83 (0.67, 1.02)	0.06
Adjusted^c^	Ref	0.83 (0.68, 1.02)	0.69 (0.56, 0.85)	0.84 (0.68, 1.04)	0.29
High-protein/cholesterol pattern	Level 1 (−3.85, −0.46)	Level 2 (−0.46, −0.13)	Level 3 (−0.13, 0.24)	Level 4 (0.24, 12.30)	
No. of metabolic syndrome	357	343	380	412	
Crude	Ref	0.93 (0.76, 1.13)	1.15 (0.94, 1.40)	1.36 (1.11, 1.68)	<0.001
Adjusted^c^	Ref	0.92 (0.75, 1.13)	1.14 (0.92, 1.41)	1.36 (1.10, 1.68)	<0.01

^a^Multiple logistic regression analysis.

^b^Adjusted odds ratios (95% confidence interval) (all such values).

^c^Adjusted for other two dietary patterns scores.

**Table 5:** Association between quartiles of food group intake and metabolic syndrome.

Food groups	Quartiles of food group intake (gram/day, n = 2984)				*P* for trend^a^
Animal food	Level 1 (0, 143.13)	Level 2 (143.13, 199.10)	Level 3 (119.10, 279.99)	Level 4 (279.99, 2092.07)	
No. of metabolic syndrome	364	362	374	392	
Crude	Ref	1.00 (0.81, 1.22)^b^	1.06 (0.86, 1.30)	1.17 (0.95, 1.44)	0.10
Adjusted^c^	Ref	1.00 (0.82, 1.23)	1.07 (0.87, 1.32)	1.21 (1.01, 1.51)	0.08
Fruits	Level 1 (0, 99.37)	Level 2 (99.37, 161.79)	Level 3 (161.79, 255.06)	Level 4 (255.06, 1743.40)	
No. of metabolic syndrome	388	364	372	368	
Crude	Ref	0.88 (0.71, 1.08)	0.92 (0.75, 1.12)	0.90 (0.73, 1.10)	0.47
Adjusted^c^	Ref	0.88 (0.71, 1.08)	0.94 (0.76, 1.16)	0.91 (0.72, 1.15)	0.67
Vegetables	Level 1 (0, 274.06)	Level 2 (274.06, 392.09)	Level 3 (392.09, 553.88)	Level 4 (553.88, 3138.11)	
No. of metabolic syndrome	405	366	337	384	
Crude	Ref	0.81 (0.66, 0.99)	0.70 (0.57, 0.86)	0.89 (0.72, 1.09)	0.29
Adjusted^c^	Ref	0.79 (0.64, 0.97)	0.67 (0.54, 0.83)	0.83 (0.65, 1.05)	0.15
Grain and grain products	Level 1 (0, 174.25)	Level 2 (174.25, 230.30)	Level 3 (230.30, 311.20)	Level 4 (311.20, 1394.72)	
No. of metabolic syndrome	373	368	379	372	
Crude	Ref	0.97 (0.79, 1.18)	1.06 (0.86, 1.29)	0.99 (0.81, 1.22)	0.86
Adjusted^c^	Ref	0.95 (0.77, 1.16)	1.04 (0.84, 1.28)	0.98 (0.78, 1.22)	0.96

^a^Multiple logistic regression analysis.

^b^Adjusted odds ratios (95% confidence interval) (all such values).

^c^Adjusted for other food groups intake.
